# Deep Neural Network for Cardiac Magnetic Resonance Image Segmentation

**DOI:** 10.3390/jimaging8050149

**Published:** 2022-05-23

**Authors:** David Chen, Huzefa Bhopalwala, Nakeya Dewaswala, Shivaram P. Arunachalam, Moein Enayati, Nasibeh Zanjirani Farahani, Kalyan Pasupathy, Sravani Lokineni, J. Martijn Bos, Peter A. Noseworthy, Reza Arsanjani, Bradley J. Erickson, Jeffrey B. Geske, Michael J. Ackerman, Philip A. Araoz, Adelaide M. Arruda-Olson

**Affiliations:** 1 Department of Cardiovascular Surgery, Cleveland Clinic, Cleveland, OH 44195, USA; dchen46@gmail.com; 2 Department of Cardiovascular Medicine, Mayo Clinic, Rochester, MN 55902, USA; bhopalwalahuzaifa@gmail.com (H.B.); nakeya52@gmail.com (N.D.); zanjiranifarahani.nasibeh@mayo.edu (N.Z.F.); sravani.lokineni@gmail.com (S.L.); bos.martijn@mayo.edu (J.M.B.); noseworthy.peter@mayo.edu (P.A.N.); geske.jeffrey@mayo.edu (J.B.G.); ackerman.michael@mayo.edu (M.J.A.); 3 Department of Internal Medicine, Mayo Clinic, Rochester, MN 55902, USA; poigaiarunachalam.shivaram@mayo.edu; 4 Department of Radiology, Mayo Clinic, Rochester, MN 55902, USA; bje@mayo.edu (B.J.E.); paraoz@mayo.edu (P.A.A.); 5 Center for the Science of Health Care Delivery, Mayo Clinic, Rochester, MN 55902, USA; enayati.moein@mayo.edu; 6 Biomedical and Health Information Sciences Department, University of Illinois, Chicago, IL 60612, USA; kap@uic.edu; 7 Department of Cardiovascular Medicine, Mayo Clinic, Phoenix, AZ 85054, USA; arsanjani.reza@mayo.edu; 8 Department of Pediatric and Adolescent Medicine, Mayo Clinic, Rochester, MN 55902, USA; 9 Department of Molecular Pharmacology & Experimental Therapeutics, Mayo Clinic, Rochester, MN 55902, USA

**Keywords:** cardiac magnetic resonance imaging, deep learning, image segmentation, hypertrophic cardiomyopathy

## Abstract

The analysis and interpretation of cardiac magnetic resonance (CMR) images are often time-consuming. The automated segmentation of cardiac structures can reduce the time required for image analysis. Spatial similarities between different CMR image types were leveraged to jointly segment multiple sequences using a segmentation model termed a multi-image type UNet (MI-UNet). This model was developed from 72 exams (46% female, mean age 63 ± 11 years) performed on patients with hypertrophic cardiomyopathy. The MI-UNet for steady-state free precession (SSFP) images achieved a superior Dice similarity coefficient (DSC) of 0.92 ± 0.06 compared to 0.87 ± 0.08 for a single-image type UNet (*p* < 0.001). The MI-UNet for late gadolinium enhancement (LGE) images also had a superior DSC of 0.86 ± 0.11 compared to 0.78 ± 0.11 for a single-image type UNet (*p* = 0.001). The difference across image types was most evident for the left ventricular myocardium in SSFP images and for both the left ventricular cavity and the left ventricular myocardium in LGE images. For the right ventricle, there were no differences in DCS when comparing the MI-UNet with single-image type UNets. The joint segmentation of multiple image types increases segmentation accuracy for CMR images of the left ventricle compared to single-image models. In clinical practice, the MI-UNet model may expedite the analysis and interpretation of CMR images of multiple types.

## 1. Introduction

Cardiac magnetic resonance (CMR) imaging is well established for the diagnosis and prognostication of many cardiac diseases [1,2]. A valuable diagnostic aspect of CMR imaging is the ability to acquire image types by the variation of sequence parameters sensitive to different pathologic changes in tissue microstructure [2]. Steady-state free precession (SSFP) sequences produce images with high contrast between the myocardium and the blood pool; this is useful for the characterization of functional and morphological findings, whereas late gadolinium enhancement (LGE) highlights tissue characteristics useful for the identification of myocardial pathologies. In clinical settings, both SSFP and LGE are acquired and utilized for the interpretation of CMR imaging of patients with hypertrophic cardiomyopathy (HCM) [3].

The analysis and interpretation of CMR images are often time-consuming. The automated segmentation of cardiac structures, such as the left ventricular wall, can greatly reduce the amount of time necessary for image analysis. A variety of methods have been proposed to automate this process, and these can be considered either non-deep-learning or deep-learning methods. Non-deep-learning methods leverage prior knowledge of the characteristics of images to determine the boundaries of anatomic structures [4,5,6,7,8]. However, most of these strictly feature-engineered methods have fallen out of favor due to the flexibility and robustness of deep learning [9]. 

Although deep-learning methods are expensive to train, the execution of a model can be performed without significant computational resources compared to iterative segmentation methods that must be performed for each new image. The most recent advances in CMR segmentation utilize combinations of prior knowledge or models and deep learning [10,11,12]. Most of these methods have focused on a single CMR image type, primarily cine SSFP, that provides a clear delineation between the blood pool and the myocardium, a high spatial resolution, and a high signal-to-noise ratio. Other methods, such as multi-sensor fusion, yield only a single mask that may be inappropriate for multi-image type segmentation where misregistration and sequence differences may require significantly different masks [13]. The multi-input fusion network (MIFNet) model is based on multi-scale input and feature fusion and showed reasonable performance with limited parameters and less training time; however, it used only SSFP sequences for segmenting CMR images [14]. The focal loss constrained residual network (FR-Net) model demonstrated improved performance in the CMR segmentation of two-dimensional (2D) short-axis CMR images; it trains the model with focal loss and dice loss alternatively [15]. A model based on an ensemble of neural networks was reported that selects the most optimal segmentation based on the predicted segmentation accuracy on the fly; it can be adapted to CMR, echocardiography, and cardiac computed tomography segmentation [16]. The BLU-Net model, which is a nested U-shape network with compressed dense blocks (CDBlocks) and dilated convolution, shows good performance on CMR segmentation based on a larger receptive field by preserving spatial resolution and feature information [17]. Although these novel methods report reasonable performance, they use one specific CMR imaging sequence dataset for model training. The motivation for multi-image type segmentation is to leverage complementary information from SSFP and LGE to provide a deep-learning solution for improving CMR imaging segmentation that saves time spent on image analysis. 

Accordingly, the goal of this study was to create a deep-learning solution that learns to jointly segment CMR images of different types to minimize the image analysis workload of radiologists. The hypothesis tested was that the joint segmentation of SSFP and LGE can provide a deep neural network architecture. We show a proof of concept of this novel model in a cohort of patients with HCM, for which CMR is routinely used both for diagnosis and for sudden cardiac death risk stratification [18,19,20].

## 2. Materials and Methods

### 2.1. Study Design

**Figure 1:**
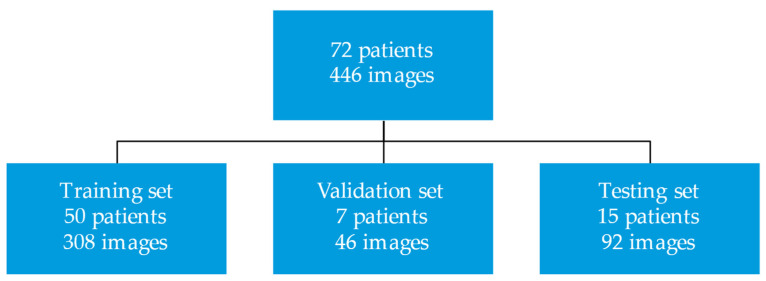
Study design.

This study was approved by the Mayo Clinic institutional review board. All patients provided informed consent for the research use of their health data. This study created a deep learning model to segment CMR SSFP and LGE images. We randomly selected 72 patients who underwent contrast CMR exams for the evaluation of HCM between 2009 and 2016 at Mayo Clinic Rochester, MN. The CMR images of these patients with HCM were divided into training (*n* = 50 patients; 308 images), validation (*n* = 7 patients; 46 images), and test sets (*n* = 15 patients; 92 images). Figure 1 displays the study design.

Standard clinical cardiac exams were conducted with short- and long-axis cine SSFP and short- and long-axis LGE in a 1.5T General Electric Optima MR450w (General Electric, Boston, MA, USA). For the segmentation, selected ventricular short-axis slices between the first appearance of the aortic valve and the disappearance of the right ventricle from the end-systolic phase of the cine SSFP and the LGE images were acquired in systole for each study. All images were extracted from the Mayo Clinic image archiving and communication system in Digital Imaging and Communications in Medicine (DICOM) format. A physician manually segmented the images for the left ventricular cavity (LVC), the left ventricular myocardium (LVM), and the right ventricle (RV), including both the myocardial wall and cavity using Radiology Informatics Laboratory Contour (RIL-Contours), which is a freely available annotation tool developed at Mayo Clinic [21]. The physician was instructed to segment ventricular slices that included all 3 structures and in which the left ventricular myocardium was not bisected by the aortic valve. The papillary muscle was included in the LVC segment. The cine SSFP images were segmented to match the cardiac frame during which the LGE images were acquired. 

### 2.2. Preprocessing

DICOM images and annotations were converted into numpy arrays using pyDicom (version 1.2), a python package used for manipulating DICOM files [22]. All images were zero-padded to a square matrix, cropped by 50% to focus on a region of interest containing the heart, and under sampled following filtering with a low-pass filter to a 128 × 128 matrix. Data augmentation with rotation between −60 and 60 degrees around the center of the image was performed to increase the training sample size and increase the likelihood of training a generalizable model.

### 2.3. Model

A multi-image type bidimensional UNet (MI-UNet) was developed to jointly learn features applicable to the segmentation of two types of CMR images: cine SSFP and LGE. This was based on the observation that multi-task learning problems have often shown improved results compared to learning single tasks individually. The network was derived from the conventional bidimensional UNet developed by Ronneberger et al. with back-to-back cascading encoder-decoder networks with skip connections [23]. In the standard UNet, each “level” consisted of two convolutional, batch normalization, and rectified linear unit activation layers in series. The encoding network used max pooling between levels to minimize feature map size, while the decoding network used deconvolution to recover the dimensions of the original input. Skip connections were also used between equivalent levels in the encoding and decoding networks. 

**Figure 2:**
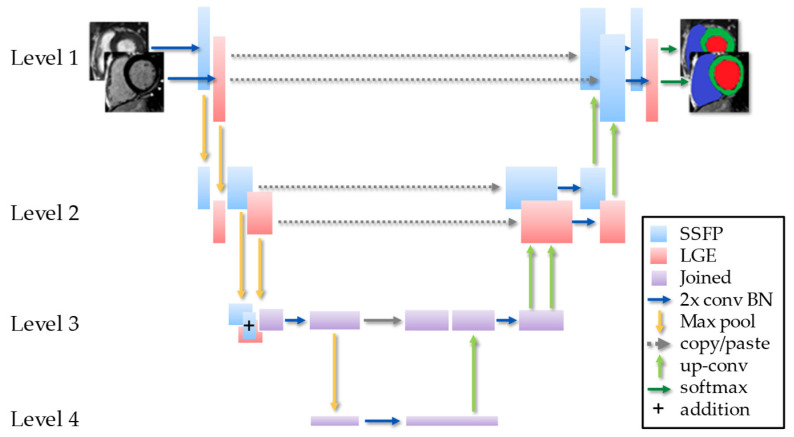
Schematic of the MI-UNet with 2 convolutions and batch normalization (2x conv BN).

In this work, rather than two independent encoder-decoder networks for each image type, the hidden layers between the two networks were additively joined and then shared at the bottom of the “U”, much like a Siamese network, as shown in Figure 2. 

The shared hidden layer that produced the optimal number of shared layers was identified. When the hidden layers were shared prior to the deepest layer, the subsequent layers acted as a single-input UNet. The final results of the two streams were not fused. Rather, the two streams shared a common deep embedding with separate encoders and decoders. This was to promote shared embedding between two spatially co-located imaging sources.

The conventional UNet utilizes a combination pixel-wise binary cross entropy (BCE) and Dice similarity coefficient (DSC) loss with empirically determined weighting for each [23,24] by Equation (1):

(1) $$L_{seg}=\lambda_{1}L_{DSC}+\left(1-\lambda_{1}\right)L_{BCE}\;$$

here $\lambda_{1}$ is the weight balancing between DSC loss and BCE loss. The DSC loss is determined by Equation (2):

(2) $$L_{DSC}\left(x,\hat{x}\right)=1-\frac{2\sum_{i}x_{i}{\hat{x}}_{i}}{\sum_{i}x_{i}^{2}+\sum_{i}{\hat{x}}_{i}^{2}}\;$$

whereas the BCE loss is determined by Equation (3):

(3) $$L_{BCE}\left(x,\hat{x}\right)=-\frac{1}{N}\overset{N}{\underset{i=1}{\sum }}{\hat{x}}_{i}\cdot log\;\left(p\left(x_{i}\right)\right)+\left(1-{\hat{x}}_{i}\right)\cdot log\;\left(1-p\left(x_{i}\right)\right)\;$$

where ***x*** is the predicted mask, $\hat{x}$ is the label, ***i*** is index for pixel, and ***p***(***x***) is the probability of the label. In the jointly trained model, the loss function needed to be applied to both the SSFP and LGE segments, and this yields Equation (4):

(4) $$L_{seg}=L_{Cine_{seg}}+L_{DE_{seg}}$$

where $L_{Cine_{seg}}$ and $L_{DE_{seg}}$ represent the loss returned by each respective branch. 

Given that we were learning to segment two anatomically similar images, we used this knowledge to further constrain the segments. First, a total variation (TV) penalty term was added for the gradient of each anatomic segment. This was accomplished a priori such that each segment was locally continuous to minimize the length of the contours around each segment. The total variation (TV) term is well approximated by an L1 norm, as follows in Equation (5) [25]:

(5) $$TV\left(x\right)=\frac{1}{N}\overset{N}{\underset{i=1}{\sum }}\left|\Delta x_{i}\right|$$

Second, we assumed that there should be minimum spatial differences between the segments of the two images. This contrast was instituted using a BCE loss between the segments of the two images. The overall loss function is as follows in Equation (6):

(6) $$L_{seg}=\lambda_{1}L_{DSC}+\left(1-\lambda_{1}\right)L_{BCE}+\lambda_{TV}\left(TV\left(x_{Cine}\right)+TV\left(x_{DE}\right)\right)\;+\lambda_{Sp}L_{BCE}\left(x_{Cine},x_{DE}\right)$$

where $\lambda_{TV}$ and $\lambda_{Sp}$ are the weights for TV and spatial BCE loss, respectively. 

### 2.4. Training

All models were trained using the training dataset, while hyperparameters were tuned using the validation dataset, and the final metrics were computed based on the test dataset. Model parameters were randomly initialized from a normal distribution, and Adam optimizer was used [26]. Models were trained using data augmentation with rotation between −60 and 60 degrees around the center of the image. For each model, the optimized hyperparameters (batch size: 2–24, learning rate: 10^−6^–10^−1^, and batch normalization) were found using a grid search maximizing DSC on the validation dataset. Specifically, for the MI-UNet model, the appropriateness of various a priori constraints (smoothness and similarity) was studied by varying the weights ($\lambda_{TV}$ and $\lambda_{Sp}$) assigned to each respective constraint. Models were implemented in python 3.6 using pyTorch on 3 Nvidia V100 32GB GPUs [27].

### 2.5. Evaluation

To evaluate performance, the proposed MI-UNet model was compared to three UNet-based architectures, namely the conventional single-image UNet (one model for each image type) and the transfer-learned UNet pretrained on one image type. The single-image UNet was trained on both cine SSFP and LGE images, and the transfer-learned UNet consisted of a conventional UNet that was first pre-trained using the corresponding imaging type. The performance of each segmentation model was evaluated using the standard Dice score (DSC) [24,28]. We used paired two-tailed t-tests at a 5% significance level to evaluate the statistical significance of the DSC of each model compared to a baseline single-contrast UNet. For the MI-UNet, the frame of the SSFP images matched the acquisition window of the LGE images. 

## 3. Results

CMR exams performed on 72 patients with a mean age of 63 ± 11 years (46% women) were included in this study. All MI-UNet models that joined encoding hidden layers provided similar DSC scores compared to the single-image type UNet for SSFP or LGE (Table 1). 

**Figure 3:**
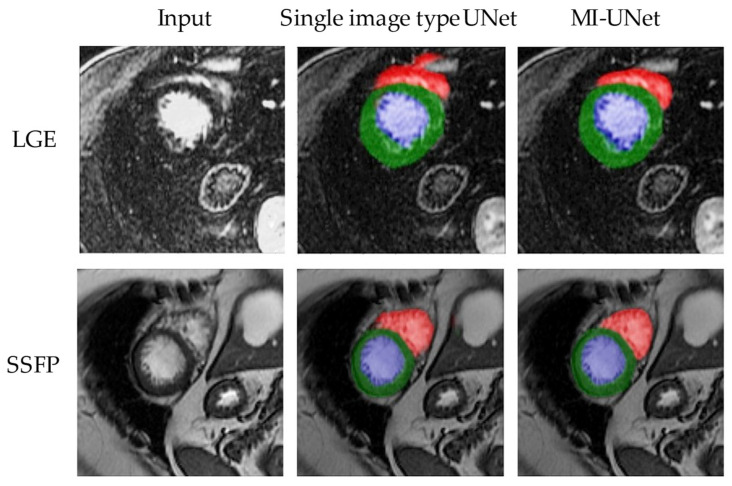
Example outputs of the segmentation models. Segments obtained from the proposed model compared with single-image type. In this figure, blue represents the left ventricular cavity, green the left ventricular wall, and red the right ventricle.

Figure 3 shows an example of segments obtained from the MI-UNet model compared with single-image type UNets. 

The MI-UNet without the use of a priori constraints ($\lambda_{TV}$ = 0 and $\lambda_{Sp}$ = 0) had a DSC of 0.87 ± 0.11 for SSFP (*p* = 0.73) and a DSC of 0.82 ± 0.16 for LGE (*p* = 0.049) when compared with a single-image type UNet. The best weights for $\lambda_{TV}$ and $\lambda_{Sp}$ were 0.3 and 0.6, respectively. Including the TV constraint and the similarity constraint individually improved the DSC scores by 0.8% and 0.3%, respectively. The two constraints used together improved the DSC scores by 2.0% with weights of 0.15 and 0.5. The MI-UNet with these constraints ($\lambda_{TV}$ = 0.15 and $\lambda_{Sp}$ = 0.5) had a superior DSC for both SSPP (*p* < 0.001) and LGE (*p* < 0.001), compared with a single-image type UNet (Table 2).

The MI-UNet achieved a superior mean DSC compared to individually trained UNets for both SSFP and LGE images. The difference across image types was most evident at the left ventricular myocardium in SSFP images and at both the left ventricular cavity and the left ventricular myocardium in LGE images. For the right ventricle, there were no differences in DCS when comparing the MI-UNet with single-image type UNets. The MI-UNet also achieved a superior mean DSC compared to transfer-learned UNets for SSFP images, with the difference across image types most evident at the left ventricular myocardium. The DSC metrics for the tested models in each segment are summarized in Table 3. The MI-UNet has 59M parameters and is 762MB in size, which is twice the size of the single-image type UNet.

SSFP—steady-state free precession; LGE—late gadolinium enhancement; LVC—left ventricular cavity; LVM—left ventricular myocardium; RV—right ventricle. 

## 4. Discussion

Novel findings from this study were the demonstration that a jointly segmented MI-UNet model improved the segmentation accuracy for both SSFP and LGE images of the left ventricle compared to conventional single-image type UNets. This technique has the potential to save time on the interpretation of CMRs by eliminating the need to manually segment different image types for the assessment of the left ventricular cavity and walls. Changes in imaging characteristics and differences in the visualization of different tissues, such as fat or flowing blood, can greatly impact the ability of conventional segmentation methods to generalize to each contrast. However, individualized segments are critically important for the accurate quantification of pathologies, such as the extent of myocardial ischemia or the distribution of fibrosis [29]. Herein, we developed a model to jointly segment cine and LGE CMR images to allow similarities implicit in the underlying cardiac anatomy to inform the segments of each image type. 

It has been widely observed that learning multiple tasks using a single network can often improve the generalizability of features, thereby improving the results for each task compared to individually learned networks [30]. There was an improvement in the cine SSFP segmentation results for the left ventricular myocardium, and we also found statistically significant improvements in the DSC scores for LGE segments both of the left ventricular cavity and the left ventricular myocardium. It appears that combining layers in the network allows the network to learn more generalizable features. We also believe that the higher signal-to-noise ratio of the cine SSFP images may provide a strong constraint for segmenting the LGE images; this is further evidenced by the efficacy of the spatial constraint offered. 

The spatial constraint offered by joint segmentation provides advantages over pre-defined spatial priors. Most importantly, spatial priors may not be well-suited for patients with hypertrophic or dilated cardiomyopathies, both of which can be associated with extensive cardiac remodeling, potentially adversely constraining segmentation. In our proposed method, rather than using a pre-conditioned shape prior, we effectively use high signal-to-noise ratio SSFP images as the prior. This constrains the LGE segments with a patient-specific prior. 

The significant improvement in LGE images is clinically important because automated quantification is utilized for the diagnosis and management of many diseases, including coronary artery disease and HCM [28,30,31], since both the extent and pattern of LGE are clinically relevant [31]. Automatic quantification of these patterns is difficult because LGE images have poor tissue delineation due to the low signal of the myocardium and the bright signal of the slow-flowing adjacent blood in the cavities [32]. Even the characteristic of myocardial enhancement varies depending on pathology; bright focal lesions are characteristic of coronary artery disease, whereas diffuse, patchy regions are indicative of fibrosis or myocardial remodeling [31]. Despite the significant difference in image characteristics, incorporating features learned from the higher signal-to-noise ratio SSFP images improves the DSC metrics of the LGE images.

A recent literature review on deep learning for cardiac imaging has shown that UNets are the most well-known and most frequently used variant of fully convolutional networks for biomedical image segmentation [33]. Several state-of-the-art cardiac image segmentation methods have adopted the UNet architecture and achieved promising segmentation accuracy [33]. Most approaches used 2D networks rather than 3D networks for segmentation due to the low through-plane resolution and motion artifacts of most cardiac magnetic resonance scanners [34].

Prior cardiac segmentation studies focused on one image type (LGE or SSFP). In contrast, the present study developed a multi-image type segmentation using both LGE and SSFP because these image types provide complementary information necessary for the diagnosis and risk stratification of hypertrophic cardiomyopathy. Additionally, the majority of the prior cardiac ventricle segmentation models for CMR were trained and tested on publicly available datasets. The present study used a clinical dataset of patients with HCM with a broad spectrum of the cardiac phenotypic characteristics of this condition [20]. A previous cardiac segmentation model was trained and tested in a dataset with only eight patients with HCM. In contrast, the present study had a larger sample size of patients with HCM, including cardiac phenotypes from real-world clinical practice. It is also important to underscore that the use of clinical datasets for the development of cardiac segmentation enables the translation of these models to clinical practice.

The motion attentive transition for zero-shot video object segmentation is a promising two-stream network structure [35]. Future experiments will compare the performance of the MI-UNet with motion attentive transition for zero-shot video object segmentation. Cross-image pixel contrast for semantic segmentation is a segmentation method that addresses intra-class compactness and inter-class dispersion [36] and will be incorporated into this segmentation framework in future experiments. Group-wise learning for weakly supervised semantic segmentation is another segmentation method that enables the discovery of relationships among groups of images [37]. This methodology will also be used in future experiments. 

There are limitations to this work. First, due to the lack of an external test set, it was not possible to evaluate model portability. Second, only two image types were evaluated, whereas the typical CMR study includes additional image types such as T1- or T2-weighted images. In the future, we plan to apply this technique to other image types, including T2- and T1-mapping. Third, we have only considered variants of the UNet for the comparison of results. In future studies, MI-Net results will be compared with SegNet [38] and ResNet [39] segmentation models. Future experiments will include additional metrics. Fourth, the dataset used for this project is unique, as it is from a cohort of patients with HCM. These patients often have variable patterns of myocardial thickening (e.g., isolated thickening of the septum) that can potentially confound atlas- and shape-based segmentation methods [40,41]. Moreover, the pattern of histopathologic fibrosis burden varies widely in the population of patients with HCM [42,43]. The inconsistencies in the presentation of HCM between the two imaging types make the segmentation task more challenging compared to a healthy population. In mitigation, the comparable performance of the segmentation model in this cohort suggests the potential for generalizability across sequences and diseases. 

## 5. Conclusions

The joint segmentation of multiple image types provides a deep neural network architecture that supports the automated segmentation of CMR images in patients with HCM. Unlike other methods that yield a single mask using multiple sensors, this method does not require a separate mask registration, which is time-consuming and prone to error. Furthermore, the method described yields superior results for left ventricular segmentation with fewer data compared to individually training separate models on different imaging types. 

## Figures and Tables

**Table 1:** Dice Similarity Coefficient for the MI-UNet compared to a single-image type UNet.

	SSFP	*p* Value*	LGE	*p* Value *
Single-image type UNet	0.87 ± 0.08		0.78 ± 0.12	
MI-UNet	0.842 ± 0.132	0.126	0.788±0.141	0.6393

SSFP—steady-state free precession; LGE—late gadolinium enhancement. * *p*-values refer to comparison with single-image type UNets.

**Table 2:** Dice Similarity Coefficients of the MI-UNet with Different Constraints.

	SSFP	*p* Value *	LGE	*p* Value *
Single-image type UNet	0.87 ± 0.08		0.78 ± 0.12	
MI-UNet $\left(\lambda_{TV}=0,\;\lambda_{Sp}=0\right)$	0.87 ± 0.11	0.73	0.82 ± 0.16	0.06
MI-UNet $\left(\lambda_{TV}=0.3,\;\lambda_{Sp}=0\right)$	0.89 ± 0.08	0.03	0.81 ± 0.20	0.21
MI-UNet $\left(\lambda_{TV}=0,\;\lambda_{Sp}=0.6\right)$	0.85 ± 0.11	0.22	0.84 ± 0.16	0.002
MI-UNet $\left(\lambda_{TV}=0.15,\;\lambda_{Sp}=0.5\right)$	0.92 ± 0.06	<0.001	0.86 ± 0.11	<0.001

SSFP—steady-state free precession; LGE—late gadolinium enhancement. MI-UNet—multi-image type UNet. $\lambda_{TV}$—is the weight for the total variation constraint. $\lambda_{Sp}$—is the weight for multi-contrast similarity constraint. * *p*-values refer to comparison with single-image type UNets.

**Table 3:** Comparison of Dice Similarity Coefficients for Tested Models in Each Segment.

MI-UNet.		Comparison with Single-Image Type UNet		Comparison with Transfer-Learned UNet	
			*p* Value		*p* Value
**SSFP**					
LVC	0.92 ± 0.06	0.91 ± 0.04	0.07	0.88 ± 0.14	0.01
LVM	0.90 ± 0.05	0.86 ± 0.07	<0.0001	0.86 ±0. 11	0.001
RV	0.88 ± 0.15	0.84 ± 0.21	0.14	0.83 ± 0.19	0.03
Mean	0.90 ± 0.07	0.87 ± 0.08	0.005	0.86 ± 0.13	0.005
**LGE**					
LVC	0.86 ± 0.12	0.78 ± 0.12	<0.0001	0.83 ± 0.14	0.11
LVM	0.89 ± 0.07	0.828 ± 0.08	<0.0001	0.86 ± 0.09	0.01
RV	0.75 ± 0.21	0.733 ± 0.21	0.56	0.69 ± 0.29	0.11
Mean	0.83 ± 0.11	0.780 ± 0.10	0.001	0.79 ± 0.15	0.04

SSFP—steady-state free precession; LGE—late gadolinium enhancement. LVC—left ventricular cavity. RV—right ventricle.

## Data Availability

The data used in this study can be made available to researchers collaborating with Mayo Clinic under a research agreement. However, the data are not publicly available due to the need to preserve the privacy of patient health information.
